# Impact of Inhomogeneous Static Magnetic Field (31.7–232.0 mT) Exposure on Human Neuroblastoma SH-SY5Y Cells during Cisplatin Administration

**DOI:** 10.1371/journal.pone.0113530

**Published:** 2014-11-25

**Authors:** Cristian Vergallo, Meysam Ahmadi, Hamid Mobasheri, Luciana Dini

**Affiliations:** 1 Department of Biological and Environmental Science and Technology (Di.S.Te.B.A.), University of Salento, 73100 Lecce, Italy; 2 Laboratory of Membrane Biophysics and Macromolecules, Institute of Biochemistry and Biophysics, University of Tehran, 13145-1384 Tehran, Iran; 3 Neuroscience Research Center, Institute of Neuropharmacology, Kerman University of Medical Sciences, 76175-113 Kerman, Iran; 4 Biomaterials Research Center (BRC), University of Tehran, 13145-1384 Tehran, Iran; Medical University of South Carolina, United States of America

## Abstract

Beneficial or adverse effects of Static Magnetic Fields (SMFs) are a large concern for the scientific community. In particular, the effect of SMF exposure during anticancer therapies still needs to be fully elucidated. Here, we evaluate the effects of SMF at induction levels that *cis*Pt-treated cancer patients experience during the imaging process conducted in Low field (200–500 mT), Open field (300–700 mT) and/or inhomogeneous High field (1.5–3 T) Magnetic Resonance Imaging (MRI) machines. Human adrenergic neuroblastoma SH-SY5Y cells treated with 0.1 µM *cis*Pt (*i.e.* the lowest concentration capable of inducing apoptosis) were exposed to SMF and their response was studied *in vitro*. Exposure of 0.1 µM *cis*Pt-treated cells to SMF for 2 h decreased cell viability (30%) and caused overexpression of the apoptosis-related cleaved caspase-3 protein (46%). Furthermore, increase in ROS (Reactive Oxygen Species) production (23%) and reduction in the number of mitochondria *vs* controls were seen. The sole exposure of SMF for up to 24 h had no effect on cell viability but increased ROS production and modified cellular shape. On the other hand, the toxicity of *cis*Pt was significantly prevented during 24 h exposure to SMF as shown by the levels of cell viability, cleaved caspase-3 and ROS production. In conclusion, due to the cytoprotective effect of 31.7–232.0 mT SMF on low-*cis*Pt-concentration-treated SH-SY5Y cells, our data suggest that exposure to various sources of SMF in cancer patients under a *cis*Pt regimen should be strictly controlled.

## Introduction

Nowadays, many industrial and medical technologies use Static Magnetic Fields (SMFs) with strong magnetic inductions (1–5 T). For example, in the aluminium industry and in particle accelerators, SMFs of 1–4 T are applied. In Magnetic Resonance Imaging (MRI) apparatus, which is for clinical diagnostic purposes, static and time-varying magnetic fields from 0.05 to 2 T are employed. However, the risks arising from human exposure to these fields have not been fully determined.

In answering this question, scientists are challenged with relating data obtained from *in vitro* and *in vivo* experiments to reveal what that occurs in humans. Many *in vivo* and *in vitro* studies concerning the biological effects of SMF-exposure, highlight both detrimental and beneficial effects. For example, meta-analysis of *in vivo* studies [1], that examined the effects of SMFs of MRI machines on humans, revealed significant impairments in various functions including: reaction time, visual processing, eye-hand coordination and working memory. However, there have been no serious side effects reported yet. Conversely, Vergallo *et al.* [2] provided *in vitro* evidence indicating that the exposure to a moderate inhomogeneous SMF for up to 24 h causes a beneficial effect on human macrophages and lymphocytes. The effects included the suppressed release of pro-inflammatory cytokines (InterLeukin (IL)-6, IL-8, and Tumor Necrosis Factor (TNF)-α) and production of anti-inflammatory cytokine IL-10.

Nevertheless, there is an increasing interest in the application of permanent magnets for therapeutic purposes. Magnetotherapy provides a safe, easy and non-invasive method to directly treat the site of injury, source of pain, inflammation, disorders and diseases [2]–[3]. Some evidence has already suggested that many cell processes can be influenced by the combined application of SMF and drugs [4]–[6]. Accordingly, the cytotoxic effect of antineoplastic drugs on cancer cells was enhanced by a combined treatment of moderate magnetic induction of SMFs and chemotherapeutic drugs. Such studies highlight the synergistic action of SMF combined with pharmacological treatment [7]–[12]. However, due to various characteristics of the field such as, induction level, duration and direction as well as the dosage of administered drug, further studies are required to reveal the mechanism(s) involved in the combined approaches.

Many different types of cancer, neuroblastoma included, are treated with an antineoplastic drug, either alone or in combination with other cytostatic and/or radiotherapeutic agents such as cisplatin (Cis-DichloroDiammine Platinum II, *cis*Pt) [13]. However, the efficacy of *cis*Pt is often accompanied by toxic side effects and tumor resistance, which in turn lead to secondary malignancies [14]. Side effects of *cis*Pt therapy involve general cellular damage, which leads to nausea and vomiting, decreased blood cell and platelet production in bone marrow (myelosuppresion) and weakened response to infection (immunosuppression) [13]. Furthermore, several specific side effects to the organs, like nephrotoxicity [15], ototoxicity, especially in children [16], neurotoxicity [17], cardiotoxicity [18] and hepatotoxicity [13] have also been reported.

Cancer patients can be subjected to MRI during chemotherapeutic treatments. MRI diagnosis requires different types of magnetic fields, with various static *B_0_* components involved [19]. Exposure to homogenous moderate SMF of 8.8 mT produced by a solenoid has shown to enhance the cytotoxic potency of *cis*Pt in human leukemic cells K562 [9], [11]. Although the magnetic induction in the imaginary defined box of the imaging area is kept homogenized, the patients experience an inhomogeneous magnetic field gradient in High Field Closed (1.5–3 T) [20], Low Field Closed (200–700 mT) [21] and Low Field Open (200–700 mT) [22] as well as in Semi-Open (350 mT) (Siemens MAGNETOM C! 0.35 T with 24 mT/m gradient; Siemens, München, Germany) MRI machines, for a maximum duration of 30–45 min. Accordingly, we tried to address the combined effect of SMF and cytotoxic effect of *cis*Pt at different magnetic induction levels that the patient is exposed during the course of MRI.

Thus, we evaluated the possible synergistic or antagonistic effects between SMF and *cis*Pt, in human adrenergic neuroblastoma SH-SY5Y cells. The cells were simultaneously treated with 0.1 µM *cis*Pt and an inhomogeneous SMF (31.7–232.0 mT) for up to 24 h. The efficiency of this approach was evaluated by means of the survival rate as well as morphological and biochemical parameters.

## Materials and Methods

### Chemicals

All chemicals were of analytical grade and provided by Sigma-Aldrich (St. Louis, MO, USA) unless otherwise indicated.

### Exposure, measurements and simulations of SMF

**Figure 1:**
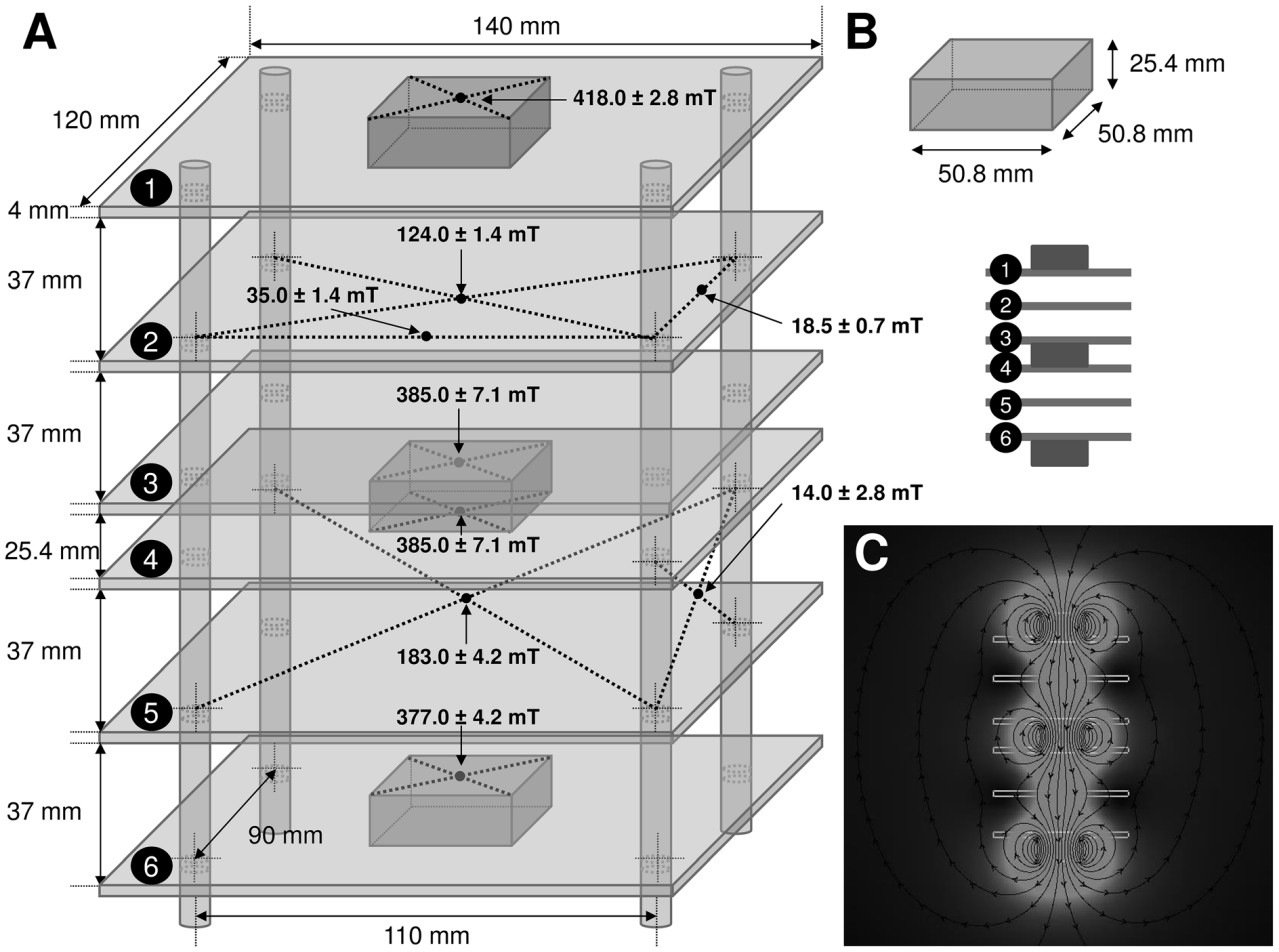
SMF exposure system. A: size and shape, not in scale, of the SMF exposure system showing the values of magnetic induction measured at different points. B: size and shape of a single magnet (not in scale) and allocation of the three magnets (not in scale). C: computer simulation in scale of magnetic flux showing the direction of the field lines. The light grey area indicates higher, while the dark grey lower values of magnetic flux density.

**Figure 2:**
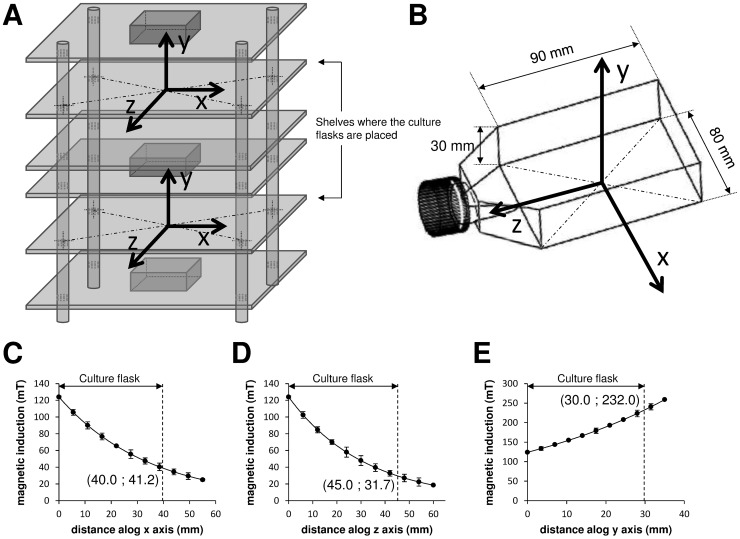
SMF exposure of cultures showing the measured and simulated magnetic inductions. A: schematic representation, not in scale, showing the exact localization where the culture flasks have been placed within the SMF exposure system. B: size and shape, not in scale, of a single culture flask. The centre of the bottom of the culture flask has been designated as the origin ‘0’ of the reference system; the x and z axes were arbitrarily chosen, and the y-axis was perpendicular to flask and outgoing up from the same flask. C-E: magnetic induction values measured (spots) and simulated (continuous black line) along the x (C), z (D), and y axes (E) of the culture flask. Each value represents the mean ± SE of six independent experiments, each done in duplicate.

SMF was produced by three magnetic parallelepipeds of NdFeB, sized 50.8×50.8×25.4 mm, coated with Ni, grade N40, *B_r_* 1260–1290 mT, magnetized through the thickness, and supplied by Webcraft GmbH (Uster, Switzerland). The magnets were kept together by attraction in a structure made of six shelves of plexiglass. The shelves of plexiglass (thickness of 4 mm) were fixed by using four bolts (8 mm of diameter) of PolyVinyl Chloride (PVC) (Fig. 1). These materials did not disturb the magnetic field configuration. Magnetic inductions were measured by using a Gaussmeter GM04 (Hirst Magnetic Instruments Ltd, Tesla House, Tregoniggie, Falmouth, Cornwall, UK), operating in the sensitivity range of 0 to more than 3 T, with 1 mT resolution and ±1% accuracy. The values and corresponding location of magnetic induction at different points of the SMF exposure system were measured (Fig. 1A), and the size, shape and location of the magnets (Fig. 1B) were considered for simulation analysis. Cell culture flasks of 25 cm^2^ (Iwaki, Tokyo, Japan) were placed in two separate SMF exposure chambers with the size of 82×102×37 mm. The modelling software Vizimag 3.193 (SoftNews Net s.r.l., Bucharest, Romania) was used to simulate the magnetic field lines distribution and to draw the magnetic flux densities within and in the vicinity of the exposure system (Fig. 1C). The configuration of the SMF exposure system and position of the culture flasks are shown in figures 2A–B. The centre of the bottom plane of the culture flask was designated as the origin ‘0’ of the reference system; the x and z axes were arbitrarily chosen to show the magnetic induction at different location on the cell culture plane (Figs. 2C–D), and the y axis to show it at different distances perpendicular to the plane (Fig. 2E). Cell cultures were always placed on the same shelves of a cell culture incubator where the ambient 50 Hz magnetic field was 0.95 and 0.62 µT with the heater on and off, respectively. The background magnetic induction in the laboratory area where the cells were processed (next to incubators, worktops and cell culture hood) ranged between 0.08 and 0.14 µT (50 Hz). There was no other significant effect, including temperature rise detected throughout 24 h incubation and exposure period. The error level in all measurements never exceeded 2%.

### Cell cultures and treatments

Human adrenergic neuroblastoma, cell line SH-SY5Y (Sigma-Aldrich), were supplemented with 10% (v/v) inactivated Fetal Calf Serum (FCS) (Biowest, Nuaillé, France), 2 mM L-glutamine (Biowest), 100 IU/ml penicillin and streptomycin in 25 cm^2^ flasks at the cell density of 10^6^ cells/ml in Dulbecco's Modified Eagles Medium (DMEM, Cambrex BioScience, Verviers, Belgium). They were then cultured in a humidified atmosphere of 5% CO_2_ at 37°C. The medium was replaced every 2 days and the cell cultures were split twice a week. Cells at about 80% of confluence were trypsinized (trypsin-EDTA, Biowest), washed and scored for viability. Culture medium was replaced with fresh one after overnight recovery and cells were categorized into four groups, each consisting of about 20×10^6^ cells. The four groups of cells marked as: i) untreated, negative control (−SMF/−*cis*Pt), ii) treated with 0.1, 0.5, or 1 µM *cis*Pt (−SMF/+*cis*Pt), iii) exposed to SMF (+SMF/−*cis*Pt), and iv) treated with *cis*Pt and exposed to SMF (+SMF/+*cis*Pt). Different treatment regimens (±*cis*Pt and/or ±SMF) were carried out for 2, 4 or 24 h, continuously. Biochemical and morphological investigations were done after each treatment.

### Microscopic analyses

#### Light microscopy

The morphology of cells was studied by an Eclipse TS100 inverted Light Microscope (LM) (Nikon, Kawasaki, Kanagawa Prefecture, Japan) throughout the 24 h course of study.

#### Electron microscopy

Ultrastructure of cells was obtained by conventional Transmission Electron Microscope (TEM) (CM12 TEM Philips, Amsterdam, Netherlands). The SH-SY5Y cells (10×10^6^) were washed with 0.2 M PBS, pH 7.4, rubber scraped and fixed with 2.5% glutaraldehyde (v/v, in 0.1 M cacodylate buffer, pH 7.4) for 1 h on ice. After an extensive washing, cells were post-fixed with 1% OsO_4_ (w/v, in 0.1 M cacodylate buffer, pH 7.4) for 1 h at 4°C. Cells were then dehydrated, embedded in Spurr's resin (TAAB Laboratories Equipment Ltd, Aldermaston, England) and examined under TEM at 80 kV.

### 3-(4,5-dimethylthiazol-2-yl)-2,5-diphenyltetrazolium bromide (MTT) assay

The percentage of viable cells was indirectly determined by MTT dye reduction. MTT is reduced by active mitochondria in living cells [23]. MTT assay was performed according to the modified method presented by Sladowski *et al.* [24]. Briefly, SH-SY5Y cells were incubated with 1 mg/ml of MTT, in DMEM, at 37°C and 5% CO_2_ for 2 h. Cells were then washed three times with Phosphate Buffer Saline (PBS) 0.2 M, pH 7.4 and the reduced MTT formazan crystals were solubilised with 1 ml of DiMethyl SulfOxide (DMSO) (Carlo Erba, Milano, Italy). The Optic Density (OD) was then read at 570 nm by a spectrophotometer (Ultrospec 4000 Ultraviolet/Visible Spectrophotometer, Pharmacia Biotech, Stockholm, Sweden).

### Nitro Blue Tetrazolium (NBT) assay

The cytoplasmic Nicotinamide Adenine Dinucleotide Phosphate (NADPH), which is produced by oxidation of glucose through the hexose monophosphate shunt, serves as an electron donor [25]. The cytoplasmatic oxidase system facilitates the transfer of electrons from NADPH to NBT and reduces NBT into formazan [25]. Thus, the NBT reaction indirectly reflects the ROS (Reactive Oxygen Species)-generating activity in the cytoplasm of cells. Briefly, cells were incubated with 335 µg/ml NBT, in DMEM, at 37°C and 5% CO_2_ for 2 h, and then washed three times with absolute methanol (Carlo Erba). The amount of diformazan salts, dissolved in 1 ml of freshly prepared 2 M KOH (Azienda Chimica E Farmaceutica, Piacenza, Italy) in DMSO solution (460 µl KOH and 540 µl DMSO), was determined by an Ultrospec 4000 spectrophotometer at 630 nm.

### Analysis of apoptosis (Western blot of cleaved Caspase-3)

Apoptosis was investigated by Western blot of cleaved caspase-3 protein of whole SH-SY5Y cells lysate. To obtain a cell lysate, at least 20×10^6^ detached cells were rinsed three times with filtered PBS 0.2 M, pH 7.4 and sonicated (Sonoplus Ultrasonic homogenizer HD 2070, Bandelin electronic, Berlin, Germany) at 40% of amplitude for four cycles, each set for 10 s followed by 5 s pause, on ice. 30 µg of protein was extracted and purified by using the ReadyPrep 2-D Cleanup Kit (Bio-Rad, Hercules, CA, USA). The sample was then denatured with 2% (w/v) Sodium Dodecyl Sulfate (SDS), 10% (v/v) glycerol, 50 mM DiThioThreitol (DTT), 1 mM PhenylMethylSulfonyl Fluoride (PMSF) and traces of bromophenol blue in 62.5 mM Tris–HCl (pH 6.8) at 95°C for 5 min. The sample constituents were separated by SDS-PolyAcrylamide Gel Electrophoresis (SDS-PAGE) in 13% T acrylamide gels at 40 mA for 2 h according to Laemmli [26]. Proteins were electrotransferred onto nitrocellulose sheet (Hybond-C extra, Amersham, UK) at 250 mA for 2 h according to Towbin *et al.* [27]. After blockage with 25 mM Tris–HCl buffer containing 3% (w/v) Bovine Serum Albumin (BSA), 127 mM NaCl and 2.7 mM KCl, pH 8.3, for 1 h, sheet was washed with TBS buffer (200 mM Tris–HCl pH 7.6 and 150 mM NaCl) containing 0.05% (v/v) Tween 20. It was then incubated with monoclonal anti-cleaved caspase-3 (17 kDa) antibody (Anti-Human Caspase-3 Antibody, MBL, Woburn, MA, USA), diluted 1∶200, at 4°C for 2 h. Specific antibody binding was detected by incubating the sheet with a goat anti-mouse Immunoglobulin G (IgG) conjugated to biotin (1∶2000 dilution) at 4°C for 2 h. The nitrocellulose sheet was then subjected to extensive washing with TBS buffer and 3-3′DiAminoBenzidine (DAB) solution for 20 min in the dark and incubated with ExtrAvidin peroxidase (diluted 1∶1500) at 4 °C for 1 h. A densitometric analysis was performed using a GS-700 Imaging Densitometer (Bio-Rad). Pre-stained low range (Bio-Rad) and biotinylated SDS Molecular Weight (MW) standards (MW range 14.3–97.0 kDa) ran in parallel to samples. Human β-actin (45 kDa) Western blots was used as control. The content of each band was quantified by using a GS-700 Imaging densitometer (Bio-Rad).

### Statistical analysis

One-way ANalysis Of VAriance (ANOVA) at the 95% confidence level was performed for all the experiments to compare six groups of data with each other. Dose-response curves were produced by the results of six groups: i) control *vs* 0.1 µM *cis*Pt-treated cells, ii) control *vs* 0.5 µM c*is*Pt-treated cells, iii) control *vs* 1 µM *cis*Pt-treated cells, iv) 0.1 µM *cis*Pt-treated *vs* 0.5 µM *cis*Pt-treated cells, v) 0.5 µM *cis*Pt-treated *vs* 1 µM *cis*Pt-treated cells, and vi) 0.1 µM *cis*Pt-treated *vs* 1 µM *cis*Pt-treated cells. In all the other cases the six groups of data were: i) control *vs cis*Pt-treated cells, ii) control *vs* SMF-exposed cells, iii) control *vs cis*Pt-treated and SMF-exposed cells, iv) *cis*Pt-treated *vs* SMF-exposed cells, v) *cis*Pt-treated *vs cis*Pt-treated and SMF-exposed cells, and vi) SMF-exposed *vs cis*Pt-treated and SMF-exposed cells. A *post hoc* Bonferroni test was performed by keeping the experiment-wise error rate at 0.05 and setting an adjustment factor of 6. Accordingly, the differences were considered to be significant at Bonferroni-adjusted critical p-value (p<0.0083 (0.05/6)). The error bars represent the mean ± the Standard Errors (SEs) of six independent experiments (n = 6), each done in duplicate.

## Results

### Experimental and computer-simulated physical measurements

SMF was produced by magnetic parallelepipeds of NdFeB arranged as shown in figures 1A–B. Two flasks were simultaneously exposed in the system. The irregular distribution of the computer simulated magnetic field lines inside and in the vicinity of the SMF exposure system are shown in figure 1C. The field lines crossing the central part of the exposure system were denser than in the peripheral area (Fig. 1C). The magnetic flux densities that depend on the characteristics of the source were inhomogeneously distributed across the plane of the culture flask. The highest flux density was identified in the centre of the magnets that decayed in a roughly linear manner towards the edges (Fig. 1C). Accordingly, the SH-SY5Y cells, which grew on the plane of the flask, were exposed to an inhomogeneous SMF with a magnetic induction ranged between 31.7 to 232.0 mT (Figs. 2C–E).

### Cell viability, morphology, cleaved caspase-3 level and ROS generation in the presence of cisPt

**Figure 3:**
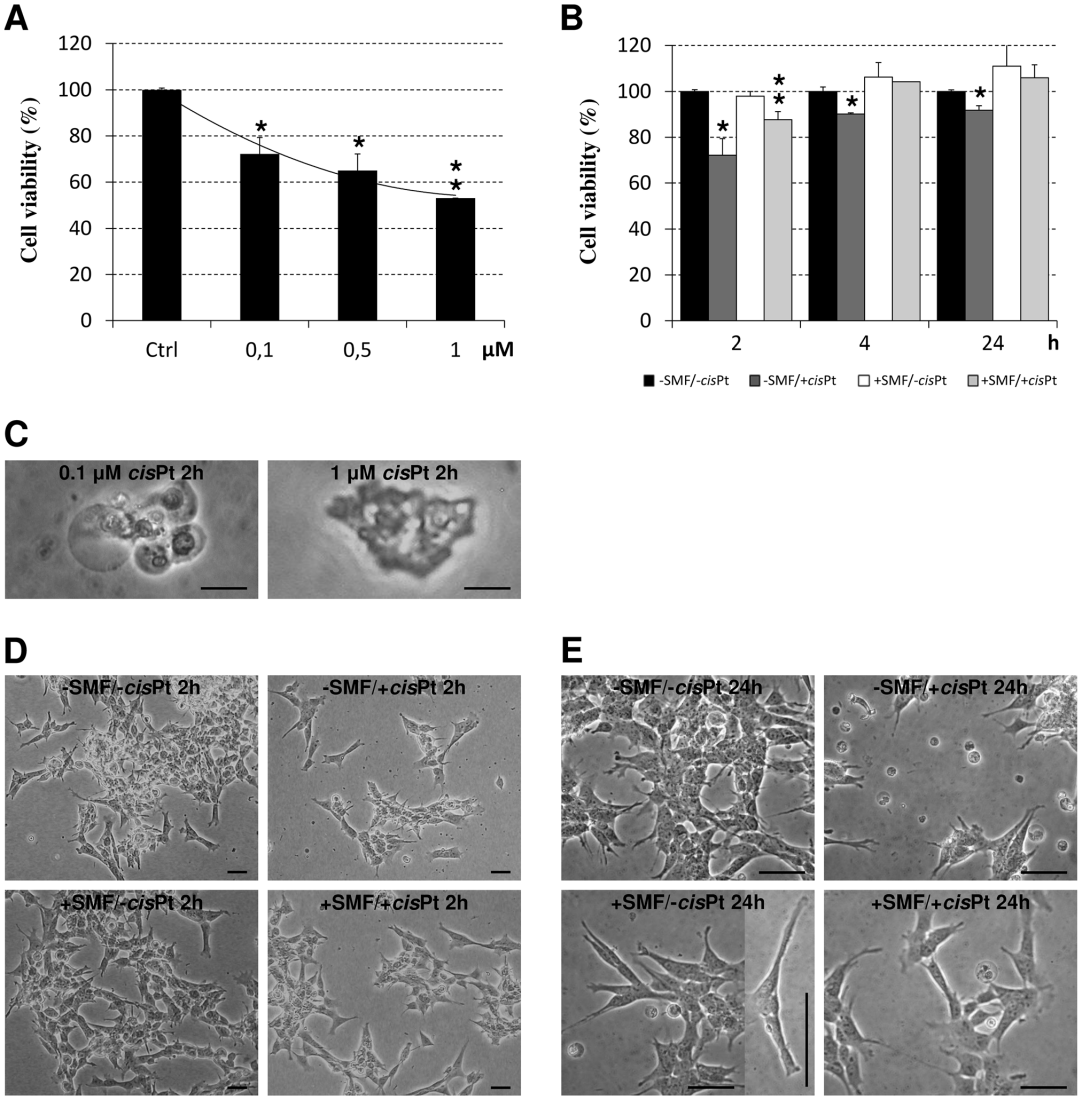
Viability and LM-based morphology of SH-SY5Y cells. A: dose-response curve of viability (MTT assay) of SH-SY5Y cells treated with different concentrations of cisplatin (*cis*Pt) (0.1, 0.5 or 1 µM) for 2 h. B: time-course of viability (MTT assay) of 0.1 µM *cis*Pt-treated and/or Static Magnetic Field (SMF)-exposed cells for 2, 4 or 24 h. C: LM images of apoptotic (left) and necrotic (right) cells respectively treated with 0.1 or 1 µM *cis*Pt. D–E: LM images of SH-SY5Y cell cultures at 2 (D) or 24 h (E) of: no treatment (−SMF/−*cis*Pt), treatment with 0.1 µM *cis*Pt (−SMF/+*cis*Pt), SMF-exposure (+SMF/−*cis*Pt) or SMF-exposure combined with 0.1 µM *cis*Pt drug (+SMF/+*cis*Pt). Values are reported as percentage of the control untreated cells considered as 100%. Each value represents the mean ± SE of six independent experiments, each done in duplicate. Single star indicates values significantly different from the respective untreated control cells (p<0.0083). Two stars indicates value significantly different either from 0.1 µM *cis*Pt-treated that from untreated control cells (p<0.0083). Bars = 10 µm.

**Figure 4:**
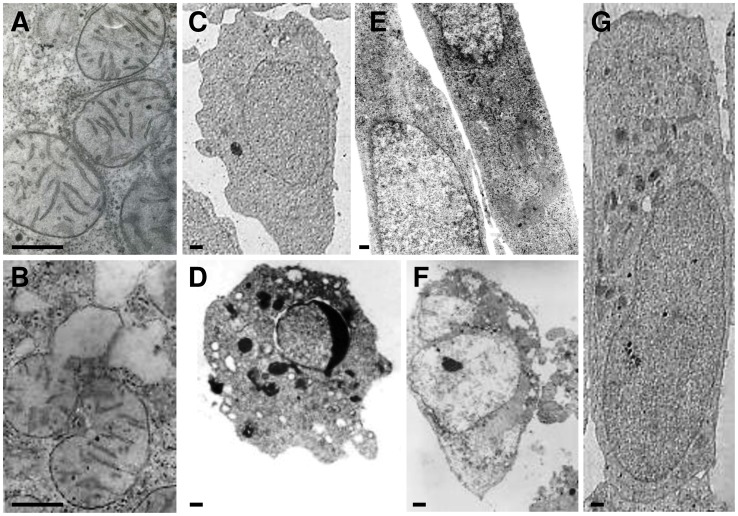
TEM micrographs of SH-SY5Y cells undergone for 2 h to different treatments. A: mitochondria of cells treated with 0.1 µM cisplatin (*cis*Pt). B: mitochondria of cells treated with 1 µM *cis*Pt. C: control cell. D: cell treated with 0.1 µM *cis*Pt (apoptotic cell). E: SMF-exposed cells. F: cell treated with 1 µM *cis*Pt (necrotic cell). G: 0.1 µM *cis*Pt-treated and SMF-exposed cell. Bars = 0.5 µm.

The cytotoxicity of *cis*Pt in SH-SY5Y cells was documented by means of dose-response curves using the data obtained through MTT assay. The *cis*Pt is an anticancer drug whose cytotoxicy is performed *via* direct DNA-damaging and consequent induction of apoptosis. Cells were cultured with increasing concentrations of 0.1, 0.5 and 1 µM of *cis*Pt, for 2 h. The percentage of cell viability in treated cells is reported with respect to the control cells (taken as 100%) (Fig. 3A). The SH-SY5Y cells were sensitive to *cis*Pt which, showed cytotoxicity in a dose-dependent manner. The number of viable cells decreased by 30% (72%, p<0.0083) in the presence of 0.1 µM *cis*Pt, 40% (65%, p<0.0083) at 0.5 µM *cis*Pt, and to about 50% (53%, p<0.0083) when incubated with 1 µM *cis*Pt for 2 h. Interestingly, based on the morphological observation of the cell culture, various concentrations of *cis*Pt induced different types of cell death after 2 h incubation. In other words, typical apoptotic features, including cell shrinkage, chromatin condensation, DNA fragmentation and extensive membrane blebbing (75±5%) were caused by 0.1 µM *cis*Pt, whereas necrosis (65±5%) was the major type of death in the cells incubated with 1 µM *cis*Pt (Fig. 3C). TEM images of apoptotic and necrotic cells are shown in figures 4D, F. An intermediate situation was observed in the cells grown in the presence of 0.5 µM *cis*Pt. Thus, the lowest concentration of *cis*Pt able to induce the highest level of apoptosis in cells was used in all time course experiments in the presence of SMF. The effectiveness of *cis*Pt was lost with time: the cell viability decreased by 10% and 8% after 4 h (p<0.0083) and 24 h (p<0.0083), respectively (Fig. 3B).

LM images showed significant changes in the morphological characteristics of different groups after 2 and 24 h of treatment (Figs. 3D–E). Further to an evident wide cell loss, the *cis*Pt-treated cells presented round shapes with highly retracted neuritis (Fig. 3D, −SMF/+*cis*Pt). Treatment with *cis*Pt dramatically damaged mitochondria, as seen in TEM micrograph (Fig. 4B). Mitochondria were scarce in the SH-SY5Y cells treated with 0.1 µM *cis*Pt for 2 h and those still present, showed damaged or totally absent *cristae* (Figs. 4A–B).

**Figure 5:**
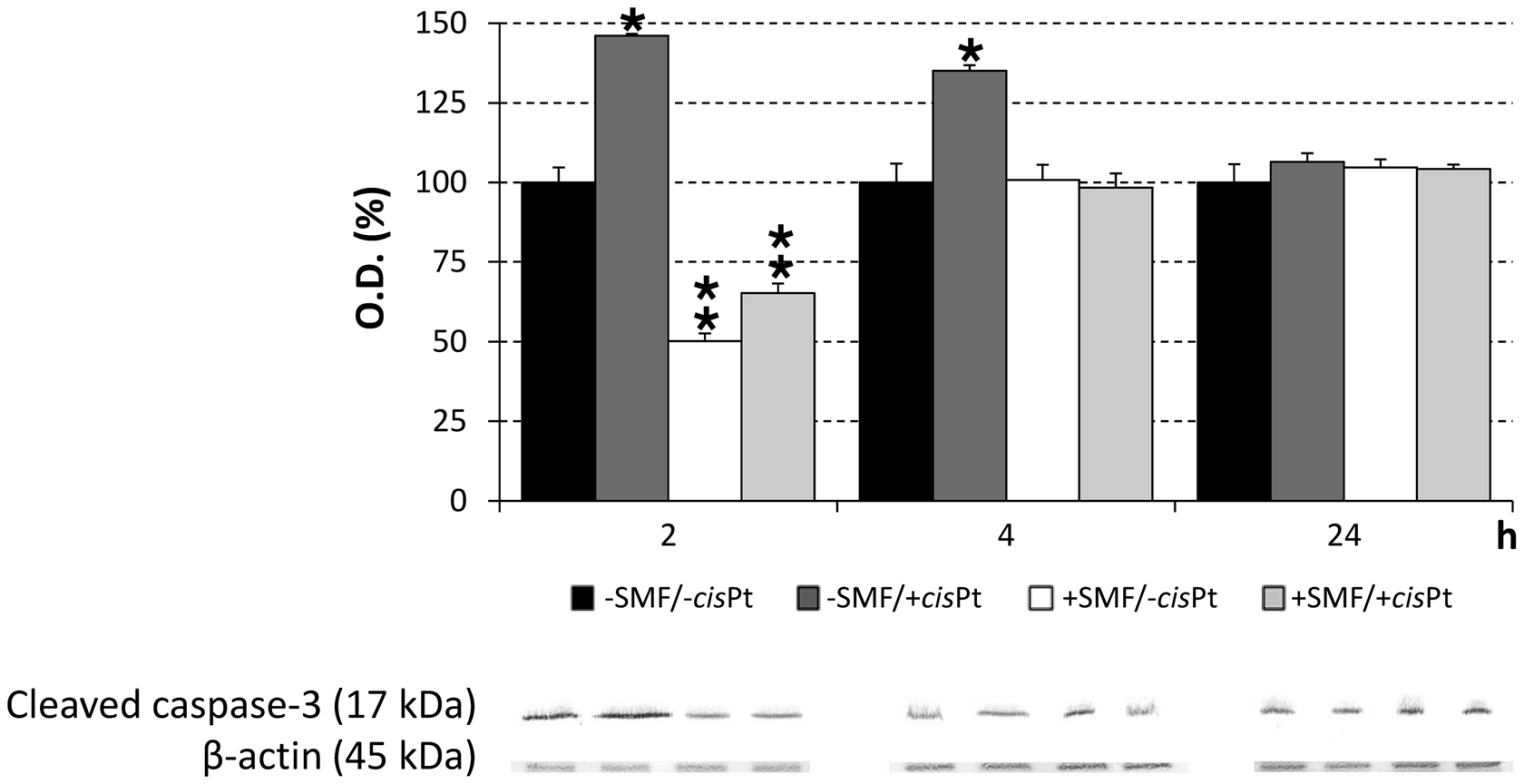
Western blot of caspase-3 of SH-SY5Y cells. Cells were exposed for 2, 4 or 24 h to different treatments: no treatment (−SMF/−*cis*Pt), 0.1 µM cisplatin (*cis*Pt) (−SMF/+*cis*Pt), Static Magnetic Field (SMF)-exposure (+SMF/−*cis*Pt) or SMF-exposure plus 0.1 µM *cis*Pt (+SMF/+*cis*Pt). The values are reported as percentage of the control untreated cells considered as 100%. Single star indicates a value significantly different from untreated control cells at the same time point (p<0.0083). Two stars indicates a value significantly different either from *cis*Pt-treated or from untreated control cells at the same time point (p<0.0083). Each value represents the mean ± SE of six independent experiments, each done in duplicate.

The apoptosis in SH-SY5Y cells was investigated by Western blot of cleaved caspase-3 (Fig. 5), the enzyme that plays a central role in the execution-phase of apoptosis. It was revealed that cleaved caspase-3 was overexpressed in SH-SY5Y cells treated with 0.1 µM *cis*Pt. The maximum level of caspase-3, 46% higher than control cells (p<0.0083), occurred within 2 h of administration of *cis*Pt. However, it decreased with time to a level 35% more than control cells after 4 h (p<0.0083) and reached a basal level equal to that in control group after 24 h of incubation.

**Figure 6:**
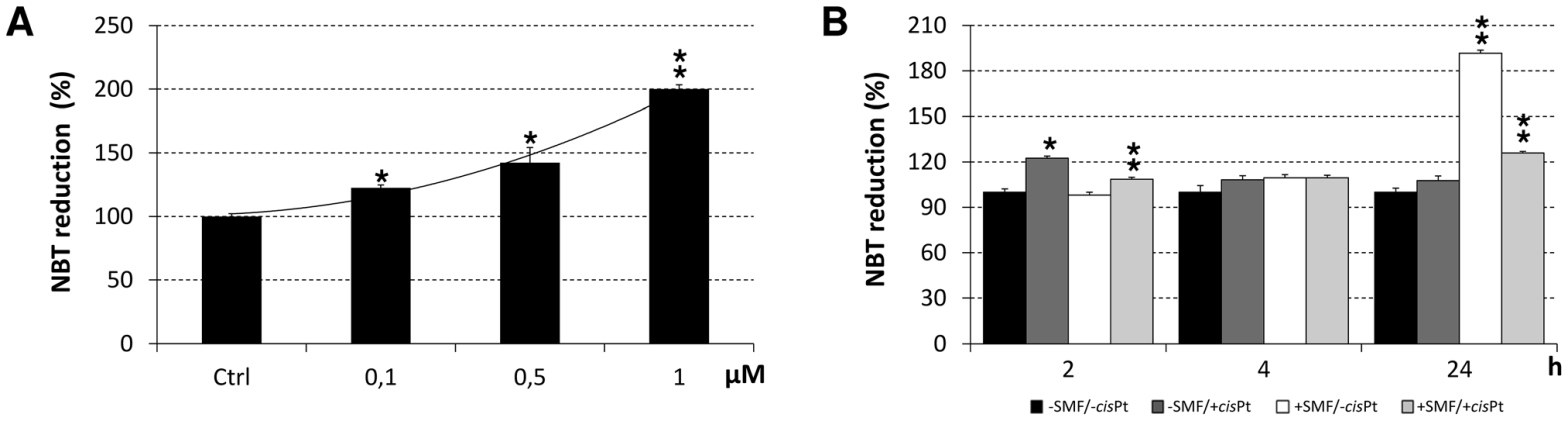
ROS production of SH-SY5Y cells. A: dose-response curve of ROS production (NBT assay) of SH-SY5Y cells treated with different concentrations of cisplatin (*cis*Pt) (0.1, 0.5 or 1 µM) for 2 h. B: time-course of ROS production (NBT assay) by 0.1 µM *cis*Pt-treated and/or Static Magnetic Field (SMF)-exposed cells for 2, 4 or 24 h. The values are reported as percentage of the NBT reduction of control untreated cells considered as 100%. Single star indicates values significantly different from the respective untreated control cells (p<0.0083). Two stars indicates value significantly different either from 0.1 µM *cis*Pt-treated that from untreated control cells (p<0.0083). Each value represents the mean ± SE of six independent experiments, each done in duplicate.

The generation of ROS molecules was increased in SH-SY5Y cells due to the induction effect of *cis*Pt molecules present in the culture medium. The concentration of ROS molecules in treated cells was indirectly evaluated by NBT assay. The extent of production of ROS molecules showed to be depended on the concentration of *cis*Pt (Fig. 6A). The maximum level of ROS, 23% higher than the control group, was identified in SH-SY5Y cells treated with 0.1 µM *cis*Pt for 2 h (p<0.0083) (Fig. 6B).

### SMF exposure effects on cell viability, morphology, caspase-3 level and ROS generation

SMF improved the life of SH-SY5Y cells during 24 h of continuous exposure. A mild increase of cell viability was found in the group that was exposed to SMF for 24 h (Fig. 3B). The unchanged values of ROS production and level of cleaved caspase-3 confirmed a positive effect of the inhomogeneous SMF. The LM observation clearly indicated: i) the lack of cell death (as shown by the cell density), ii) the absence of apoptotic and necrotic cells and iii) extensive changes in the cell shape (Fig. 3E). The morphology of SH-SY5Y cells was monitored during the course of the experiments, using phase contrast and Differential Interference Contrast (DIC) microscopy. The SH-SY5Y cells grew as clusters of neuroblastic cells with multiple, short, fine processes (neurites). Cultures grew to high density as multilayers with numerous cell clumps. Under phase-contrast and TEM microscopy, the SH-SY5Y cells showed a basic neuronal morphology, an elongated shape with a limited number of neurites of medium length, intercellular connections and neurite branching (Figs. 3–4). After 24 h exposure to SMF the SH-SY5Y cells presented enlarged cell bodies and reduced branching, but as a whole, they were elongated, mainly due to the formation of longer neurities (Fig. 3E).

### SMF exposure effects on viability, morphology, level of cleaved caspase-3 and production of ROS in the cisPt-treated cells

Exposure of the SH-SY5Y *cis*Pt-treated cells to SMF during 24 h incubation, prevented the *cis*Pt-induced toxicity in a time-dependent manner (Figs. 3–4), thus antagonizing the *cis*Pt chemotherapy efficiency. Data obtained from MTT assay and morphological observations gave comparable results (see LM images in figure 3 and TEM images of figure 4). The negative effects of *cis*Pt on the morphology of SH-SY5Y were reduced in the cells, which were treated with *cis*Pt and exposed to SMF, simultaneously. Cells maintained their neuronal shape with a discrete presence of branching neuritis whose length was comparable, but thicker than those in the control group, after combined treatment for 24 h. The ameliorative effects of SMF on *cis*Pt-treated cells were confirmed by the almost recovered number of mitochondria and morphology of *cristae*, one of the main morphological toxic outcomes of the *cis*Pt effects (data not shown). In addition, the exposure of cells to SMF reversed the ROS generation induced by 0.1 µM *cis*Pt after 2 and 4 h incubation (Fig. 6B). A significant increase in the ROS production (92% more than control cells, p<0.0083) was observed when cells were exposed to SMF for 24 h (Fig. 6B). At shorter simultaneous *cis*Pt and SMF treatment time (2 h), the amount of cleaved caspase-3 was reduced to about 65% of untreated cells (p<0.0083), but, was still significantly more than the amount measured in cells treated only with SMF (50% more than control).

## Discussion

In this study, effective protection of inhomogeneous SMF (31.7–232.0 mT) against *cis*Pt toxicity in human adrenergic neuroblastoma SH-SY5Y cells is reported. In an *in vitro* approach, we aimed to simulate and mimic the condition that cancer patients treated with *cis*Pt experience under exposure to the magnetic field of MRI.

Accordingly, the magnetic induction levels applied in the present work were chosen within the range of the static *B_0_* component used for the MRI diagnosis (0.05–2 T). It is worth noting that the extent of magnetic induction differs in various parts of the human body due to the inhomogeneous gradient of magnetic fields induced by the magnet, out of the homogeneous imaginary imaging cube that is defined by the operator. Furthermore, Time-Varying-Gradient magnetic fields (dB/dt) are used at different ramp duration to stimulate heart, brain and certain parts of the nervous system [20]. The extent of the magnetic field gradient varies in different MRI machines, thus, the patient experiences a wide spectrum of inhomogeneous magnetic fields in and/or in the vicinity of the magnetic sources. The magnetic field in various MRI machines differs, ranging from 200–700 mT in Open Low Feld MRI machine to several Tesla in Closed High Field ones [22]. Consequently, some parts of the patient's body are always exposed to an inhomogeneous magnetic field with a strength of zero to several Tesla based on their location and distance from the core of the magnet.

Different biological effects of SMF have been reported at different induction levels, however, no direct correlation and mechanism has been approved yet [2], [5]–[6], [28]–[33]. Here, we address the effect of 32–120 mT inhomogeneous magnetic fields on the *cis*Pt-treated SH-SY5Y cells under conditions that mimic the situation in MRI treated cancer patients to some extent. There is no doubt that the matter should be further addressed at different magnetic induction ranges implemented by different MRI machines. However, the significant results obtained here, indicate the appropriateness of the applied range in the reduction of *cis*Pt toxicity. Magnetic fields with low inductions have shown significant effects on cancer cells and patients [34]; this is the reason why we chose the moderate induction levels to address the biological effects of inhomogeneous SMF. The SMF system used here has an advantage because cell cultures were treated only inside the SMF induction area, where the magnetic strength at each point in the allocated space was well defined (41.2–20.0 mT along x axis, 31.7–120.0 mT along z axis and 120.0 mT along y axis).

In agreement with other reports, we found that *cis*Pt is an efficient inducer of cell death for the SH-SY5Y cells that imposes its cytotoxicity in a concentration dependent manner [35]. Moreover, our results are consistent with the findings of Lieberthal *et al.* [36] on mouse proximal tubular cells, indicating that the type of cell death (*i.e.*, apoptosis or necrosis) is also dependent on the concentration of the administered drug. Cell death occurred via apoptosis 2 h after administration of 0.1 µM *cis*Pt, whereas necrosis was the cause of their death when they were treated with higher concentration of drug, *i.e.* 1 µM *cis*Pt, at the same incubation time. Execution of the apoptotic pathway is driven by specific caspases (caspases 3 and 7). Thus, besides the morphological observation, in the presence of 0.1 µM *cis*Pt, the overexpression of cleaved caspase-3 protein, by 46% and 35% that was identified after 2 and 4 h incubation, respectively, confirmed the onset of apoptosis. The decrement of the efficacy of the *cis*Pt, *i.e.* cleaved caspase-3 overexpression diminish with time, is in agreement with the fact that *cis*Pt degrades with time [37]. It is most likely that apoptosis, in our system, is executed through the mitochondrial pathway, as the occurrence of mitochondria damage after *cis*Pt incubation strongly suggest. The mitochondrial pathway of apoptosis is known to be the main pathway used by the SH-SY5Y cells when they were treated with other anticancer drugs [38]. However, to date, the full definition of the mechanism by which *cis*Pt induces apoptosis in these cells is still under debate. It could be hypothesized that P21 Activated Kinase-2 (PAK-2), that has a dual function in the regulation of cell survival and cell death, is cleaved by cleaved caspase-3. To a lesser extent it can also be cleaved by active caspases 8 and 10 to generate the constitutively active PAK-2p34 fragment which leads cells to die by apoptosis [39]. The cell death response is induced after the translocation of the activated PAK-2p34 fragment into the nucleus [40]. Interestingly, it has been found that caspase-3 activation of PAK-2, by generating the active PAK-2p34 fragment, also occurs during *cis*Pt-induced apoptosis of the SH-SY5Y cells [41]. However, the contribution of ROS in the induction of apoptosis and/or cell death, has to be considered too. Indeed, a significant increment in ROS generation (about 23% over the control) as well as the severe decline in cell viability (almost 30%) was identified after incubation with 0.1 µM *cis*Pt for 2 h. Thus, in this case, ROS molecules are responsible for the cell loss observed in the first 2 h of treatment with *cis*Pt and also for the decline in the number of mitochondria and the damage occurred in mitochondrial *cristae*. In this scenario, the biological effects of SMF need to be further investigated. Since the lower concentration of *cis*Pt used in this study was able to mainly induce apoptosis, this concentration was chosen to investigate its biological effects in *cis*Pt-treated cells in the presence of SMF. It is already known that the SMF does not *per se* interfere with the rates of proliferation and spontaneous apoptosis in different cell lines. It can induce modulation of cell death responses, in particular, when it is combined with simultaneous administration of drugs [5]–[6]. To further reveal the complexity of the system, it should also be noted that the death response is related to the physiological condition of the cells, e.g. normal *vs* transformed *vs* undifferentiated [5]. In agreement with the above findings, the viability and level of cleaved caspase-3 in SMF-exposed cells were comparable to that in negative control.

Longer SMF exposure time induced ROS generation and increased it by about 100% that is consistent with the recent findings reported by Calabrò *et al.* [42]. This effect is probably due to the exposure to SMF and induction of conformational changes in antioxidant enzymes that causes them to lose their catalytic activity [43]. Interestingly, the effects of SMF on the exposed cells can be correlated with the morphological changes occurring at this stage of exposure. Indeed, cells were dramatically elongated and neurosomes substantially enlarged. One possible explanation for these morphological changes is that SMF could affect various aspects including, the lifetime of radical pairs, yields of caged products, and rate of released materials. In cases where the SMF affects the cells by means of interference with the radical pair mechanism, the spin of electrons in free radicals is changed. This may lead to changes in the kinetics of chemical reactions and possibly alter cellular function [44]. In fact, SMF inductions, even at levels as low as 6 mT SMF, were able to modify cell shape [28] as a result of cytoskeletal rearrangements [31] and/or through direct influence on the structural components of the plasma membrane [32].

The efficacy of *cis*Pt on the SY-SH5Y cells was significantly reduced when cells were treated with the drug and SMF, simultaneously. In fact, the viability was increased up to about 15% in combined *vs* the sole *cis*Pt-treated cells, while the level of cleaved caspase-3 and ROS production were reduced by about 80% and 18%, respectively. The protective effect of the SMF against the action of the *cis*Pt that was revealed in this study is in agreement with other studies that report protection against drug-induced apoptosis in different cell lines and primary cultures [5]. On the other hand, taking similar approaches in one [5], and different types of cells [10]–[12], [45]–[46], it was shown that drug activity was enhanced by SMF exposure. The synergistic effects of SMF with certain drugs, including *cis*Pt, taxol, doxorubicin and cyclophosphamide have been already reported [11]. However, the involvement of a variety of mechanisms has made the matter dependent on the nature of the drug, medium, and the characteristics of the applied field. Tenuzzo *et al.* [5] have reported that different responses of normal stabilized or transformed cells to the 6 mT SMF is due to the different electrical behaviour of tumour and normal cells. The group of Qi attributed the enhanced activity of different anticancer drugs on K562 cells, to the capacity of the SMF in creation of holes in the plasma membrane, thus, facilitating the drug internalization [10]–[12].

The magnetic field imposes its effect in an agonistic or antagonistic manner, depending on induction, duration, and physicochemical condition of the microenvironment. These include: i) pH by carbon dioxide hydration [47]–[48]; ii) nanobubble formation [49]; iii) zeta potential [49]; iv) ionic strength and salt mobility that act oppositely at high and low salt concentrations [50]; and v) decrease [51] and increase [8] in membrane permeability. Thus, the matter should be addressed per case, according to the status applied.

Here, on the basis of the data reported by Peleg-Shulman *et al.* [52], it should be hypothesized that the SMF affects the drug hydration status, making it unavailable and/or inactivated through hydration, accumulation, crystallization, and sedimentation processes. In other words the drug did not act synergistically with SMF, but acted in an antagonistic manner. The effectiveness of *cis*Pt on the target cells depends on the extent of its interaction with the constituents of the target cell in particular DNA molecules. Encapsulation of *cis*Pt in liposomes and evaluation of its oxidation state, chemical shift anisotropy, hydration state and interaction with the phospholipid by ^195^Pt NMR spectroscopy, has shown that *cis*Pt precipitates can be considered unavailable and ineffective so that only the soluble portion of the drug within the liposome could interact with cellular DNA [52]. These findings are further supported by the Extended X-Ray Absorption Fine Structure (EXAFS) method analysis of *cis*Pt encapsulated in liposomes that proposes a supersaturated state for *cis*Pt in the liposomes instead of the hydrated one [53]. Similar to our approach, *cis*Pt is normally administered in the presence of FCS, BSA or Human Serum Albumin (HSA) where, due to the specific important binding sites in albumin molecules, the chance of crystallization of *cis*Pt is very low [54]. Thus, the protective effect of SMF, which was identified in the current study, might be due to the facilitated crystallisation of *cis*Pt molecules caused by the long lasting exposure of treated cells to SMF. This is consistent with the effects of SMF on water that have been shown to cause strengthening hydrogen bonds at 200 mT [55] and ordering water structures around the hydrophobic molecules and colloids [56]. In other words, SMF may have changed the hydration status of the *cis*Pt molecules to a more hydrophobic state and caused them to aggregate, become unavailable, and ultimately inactivate molecules, thus exerting less cytotoxicity.

In conclusion, the overall data reported here give two contradictory indications. On one hand the data suggest that inhomogeneous SMF with a magnetic induction of 31.7–232.0 mT protects the exposed cells against *cis*Pt cytotoxicity, at least for up to 24 h. However, in the perspective of cancer patients undergoing chemotherapy this effect is certainly undesired. In other words the exposure to SMF could compromise the success of chemotherapy in *cis*Pt-treated patients, due to the decline in the effectiveness of the drug as an apoptotic agent. On the other hand, SMF could be exploited to counteract the cytotoxic effects of *cis*Pt, diffuse away, and accumulate at low concentrations in healthy tissues of patients. The penetration of *cis*Pt into the central nervous system does not occur readily; thus, a significant amount of drug is trapped in intracerebral tumour and oedematous brain tissues adjacent to the tumour site [57]–[58].

Consequently, the beneficial counteracting effects of SMF, on *cis*Pt found in our *in vitro* study might be considered in clinical therapy for prevention or reduction of side effects of the *cis*Pt-based chemotherapy.
